# Prevalence and Histopathological Characteristics of *KCNJ5* Mutant Aldosterone-Producing Adenomas in a Multi-Ethnic Malaysian Cohort

**DOI:** 10.3389/fendo.2019.00666

**Published:** 2019-10-04

**Authors:** Syahirah Kaja Mohideen, Muaatamarulain Mustangin, Nor Azmi Kamaruddin, Rohaizak Muhammad, A. Rahman A. Jamal, Norlela Sukor, Geok Chin Tan, Elena Aisha Azizan

**Affiliations:** ^1^Department of Medicine, The National University of Malaysia (UKM) Medical Centre, Kuala Lumpur, Malaysia; ^2^Department of Pathology, UKM Medical Centre, Kuala Lumpur, Malaysia; ^3^Department of Surgery, UKM Medical Centre, Kuala Lumpur, Malaysia; ^4^UKM Medical Molecular Biology Institute, UKM Medical Centre, Kuala Lumpur, Malaysia

**Keywords:** aldosterone-producing adenomas, *KCNJ5*, primary aldosteronism, Malaysia, Asia

## Abstract

Studies on excised adrenals from primary aldosteronism patients have found that somatic mutations in *KCNJ5* frequently cause excess aldosterone production in the culprit aldosterone-producing adenoma (APA). *KCNJ5* mutant APAs were reported to be peculiarly overrepresented among young females and in Oriental cohorts, compared to their older male, or Caucasian counterparts. These larger APAs were also reported to have similarities with the zona fasciculata (ZF) in the adrenal both from the steroid production profile and the morphology of the cell. We therefore aimed to corroborate these findings by characterizing the APAs from a multi-ethnic Malaysian cohort. The prevalence of *KCNJ5* mutations was estimated through targeted DNA sequencing of *KCNJ5* in 54 APAs. Confirmation of APA sample acquisition was performed by CYP11B2 immunohistochemistry (IHC) staining. The ZF steroid production profile was based on the ZF enzyme CYP17A1 IHC staining, and ZF cell morphology was based on a high cytoplasm to nucleus ratio. Seventeen (31.5%) APAs studied, harbored a *KCNJ5* mutation. No female over-representation was seen in this cohort though females were found to have a higher expression of CYP11B2 than males (*p* = 0.009; Mann-Whitney *U* test). Age at adrenalectomy correlated negatively with the percentage of ZF-like cells in the APA (*p* = 0.01; Spearman's rho) but not with the *KCNJ5* genotype. *KCNJ5* mutant APAs had a high percentage of ZF-like cells (and high CYP17A1 expression) but so did the wild-type APAs. In summary, prevalence of *KCNJ5* mutant APAs in this cohort was similar to other Caucasian cohorts, however, over-representation of females did not occur, which is similar to some studies in Oriental cohorts.

## Introduction

Primary aldosteronism (PA) is the most common cause of secondary hypertension, with an estimated prevalence of ~10% among hypertensive patients and ~20% among resistance hypertension (1–6). The presence of an autonomous aldosterone-producing lesion causes uncontrolled production of aldosterone which under normal circumstances is regulated by the renin-angiotensin-aldosterone system. Unilateral aldosterone-producing adenomas (APA) in the adrenals and bilateral adrenal hyperplasia (BAH) are major causes of the occurrence of PA, accounting for ~95% of all PA patients (7). Patients diagnosed with a unilateral APA can be surgically cured of their PA by adrenalectomy which can also clinically cure 33–77% of hypertension in these patient cases (8–10).

Studies performed in excised APA tissues found functional somatic mutations in *KCNJ5, ATP1A1, ATP2B3, CACNA1D*, and *CTNNB1* cause PA (11–15). These aldosterone-driver mutations have also been found in aldosterone-producing cell clusters (APCC) in normal adrenal glands (16) and in micronodular lesions (17). Surprisingly, the gene most frequently mutated in APAs, *KCNJ5*, is frequently of the wild-type in APCCs and micronodular lesions (16–20). The *KCNJ5* gene encodes for the G-protein-activated inward rectifier K^+^ channel 4, GIRK4. The two most common somatic mutations in *KCNJ5* are the G151R and L168R mutations located in or near the selectivity filter of this K^+^ channel (13). Presence of these mutations causes loss of the K^+^ channel selectivity leading to increased Na^+^ conductance, cell depolarization, and thus autonomous aldosterone production.

Peculiarly, most studies documenting the prevalence of causal somatic mutations in PA patients found *KCNJ5*-mutated APA commonly occurs in females which were also more frequently on the large side of the APA spectrum and had a zona fasciculata (ZF)-like steroid production profile and cell morphology (18–25). To note, even aldosterone- and cortisol-co-secreting adrenal adenomas have been found to have *KCNJ5* mutations (26). Of further interest, a higher prevalence of *KCNJ5* mutation were found in Oriental cohorts when compared to Caucasian cohorts. More than 50% of the APAs studied in cohorts from China, Japan, Korea, Taiwan, and Thailand were reported to have a *KCNJ5* somatic mutation (23, 27–33). A meta-analysis study performed on available studies at the time, estimated that the prevalence of the *KCNJ5* mutation in APAs from Oriental cohorts were almost twice than that in Caucasian cohorts (63 vs. 35%) (34).

In this study, we therefore aim to interrogate the prevalence and histopathological characteristics of *KCNJ5* mutant APAs, from a multi-ethnic Malaysian cohort in a single tertiary center.

## Materials and Methods

### Recruitment

The medical records of patients who had undergone adrenalectomy at the National University of Malaysia Medical Center between 2000 and 2015 were taken from either the CT scan report, adrenal vein sampling report, or histopathology report. Patients who had undergone adrenalectomy due to PA and who had archived FFPE adrenal samples were consecutively recruited for the study. Fifty-four confirmed APAs had sufficiently good quality material for immunohistochemistry and genetic analyses. Follow-up clinical data was available for 29 patients. The protocol used in this study has been approved by the local research ethics committee of the National University of Malaysia Medical Center.

### Immunohistochemistry (IHC) Staining

**Figure 1:**
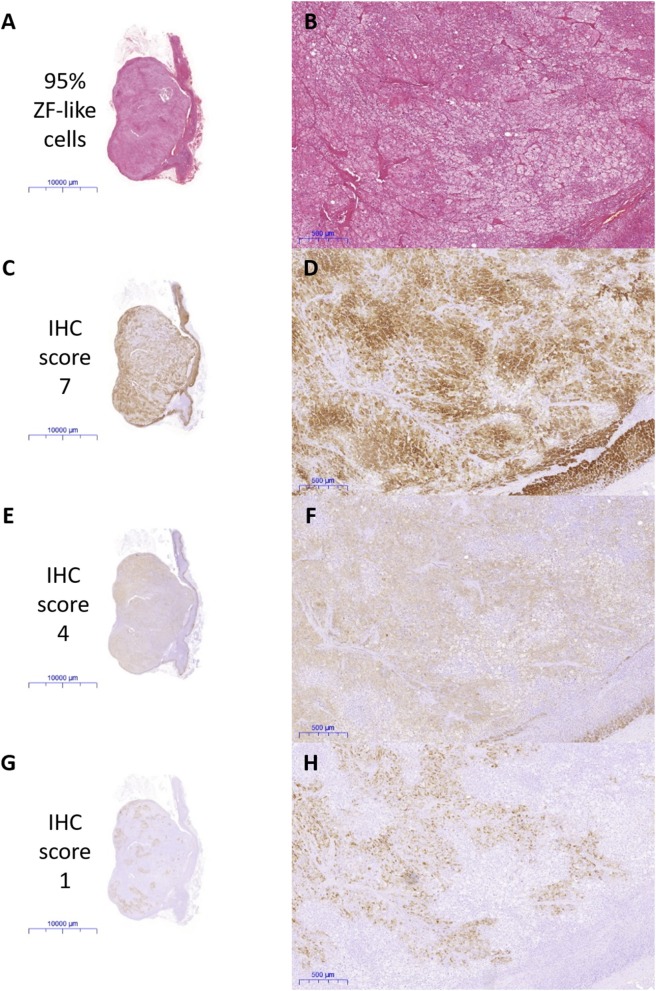
Representative scan and microscope images of the immunohistochemical (IHC) staining scored by a blinded histopathologist. **(A,B)** Percentage of ZF-like cells, **(C,D)** IHC staining of CYP17A1, **(E,F)** IHC staining of *KCNJ5*, and **(G,H)** IHC staining of CYP11B2.

**Figure 2:**
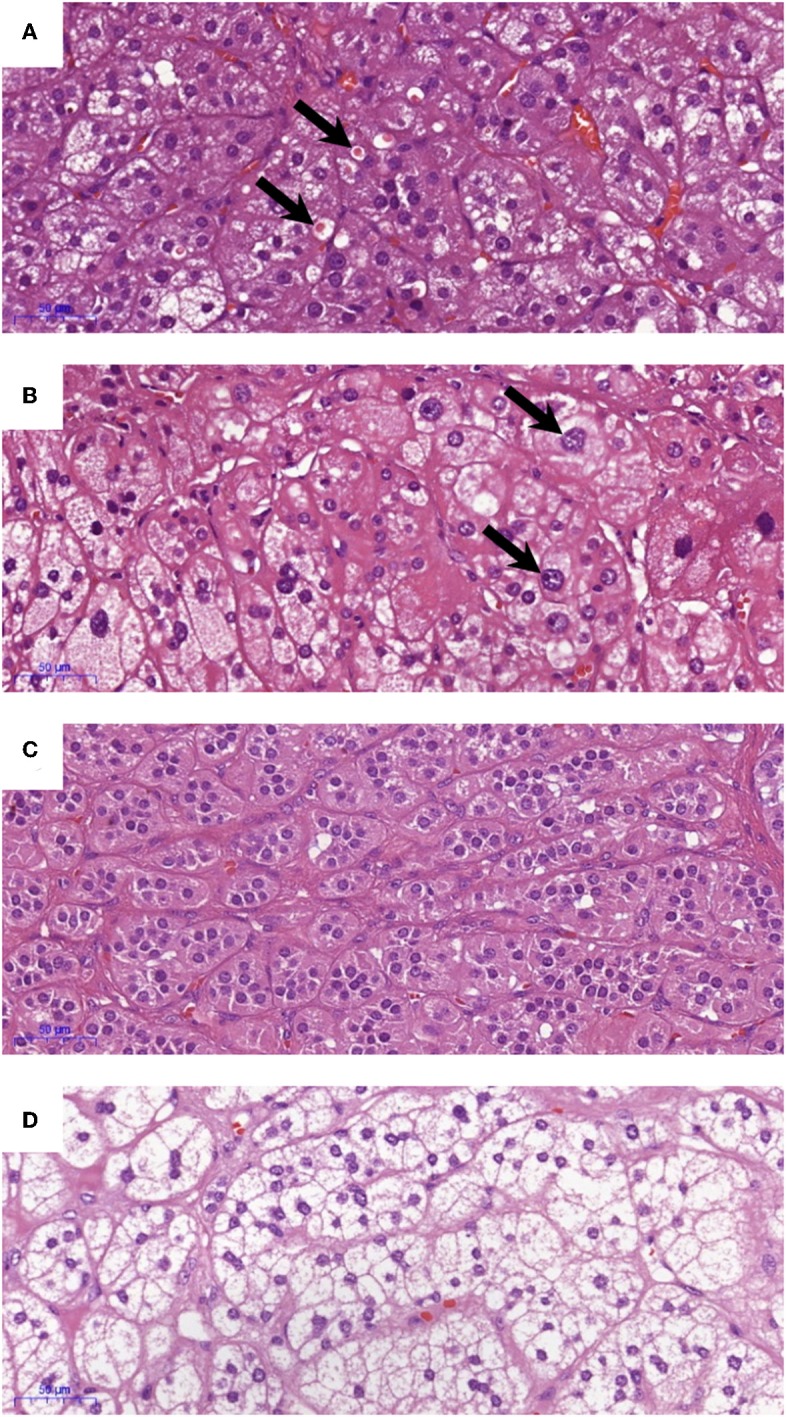
Examples of histopathology morphology based on hematoxylin and eosin (H&E) staining. **(A)** Spironolactone bodies, **(B)** atypical cells, **(C)** ZG-like cells, and **(D)** ZF-like cells.

All IHC staining was performed on 4 μm formalin-fixed paraffin embedded sections of adrenals. CYP11B2, CYP17A1, KCNJ5, and active caspase 3 staining was performed as detailed in the Supplementary Methods. Positive control tissues were used to optimize the IHC protocol (optimized results shown in Supplementary Figure 1). Negative controls where the primary antibodies are omitted were performed for all IHC experiments. IHC staining for CYP11B2 was performed on all FFPE blocks that were available of the excised adrenals, to confirm sampling of an aldosterone-producing lesion. Sections that had a positive nodule with CYP11B2 were then stained with CYP17A1, *KCNJ5*, and active caspase 3 and scored. Percentage of cells in the APAs with ZF-like cell morphology (high cytoplasm: nucleus ratio), percentage of atypical cells, and counts of spironolactone bodies were determined using hematoxylin and eosin (H&E) stained sections. All analyses of IHC and H&E staining were performed by a histopathologist blinded to the genotype results. Primary antibodies and parameters used for IHC staining are described in Supplementary Table 1, and the scoring table used for the IHC staining is detailed in Supplementary Table 2. Representative images of the IHC staining scores are shown in Figure 1. Examples of histopathology morphology based on H&E staining are shown in Figure 2.

### Genotyping of *KCNJ5* in APA DNA Samples

DNA samples of APAs were extracted from FFPE tissue blocks or FFPE sectioned slides using the commercially available kit ReliaPrep FFPE gDNA Miniprep System (Promega, USA) according to the manufacturer's instructions. The DNA sequence encoding the selectivity filter region in *KCNJ5* was amplified using the AmpliTaq Gold™ Fast PCR Master Mix (ThermoFisher Scientific, USA) according to the manufacturer's instructions. The primers and parameters used are listed in Supplementary Tables 3 and 4. PCR products were then Sanger Sequenced commercially (Apical Scientific Sdn Bhd, Malaysia) and any mutations were confirmed through sequencing of a replicated PCR product in the opposite direction.

### Statistics

Results are expressed as mean ± standard deviation (SD) unless specified otherwise. Normally distributed datasets were compared using the two-tailed *t*-test, whereas non-normally distributed datasets were compared using the Mann-Whitney *U* test. Dichotomous categorical datasets were compared using Fisher's Exact test. Relationships between variables were tested either using the Pearson's Correlation for normally distributed datasets or using Spearman's rho for non-normally distributed datasets. Normal distribution of datasets was tested using the Shapiro-Wilk test and the homogeneity of variances was tested using the Levene's test. A *p* < 0.05 was considered statistically significant. Statistical analyses and graphs were performed using IBM SPSS Statistics (version 25).

## Results

### Prevalence of *KCNJ5* Mutant APAs in a Multiethnic Malaysian Cohort

Genetic analysis identified 17 patients with a *KCNJ5* mutant APA (Table 1). Fourteen patients had a G151R *KCNJ5* mutation, while three had a L168R *KCNJ5* mutation. There was no overrepresentation of females with *KCNJ5* mutant APAs and there were no significant differences between the number of Malay patients and the number of Chinese patients that had a *KCNJ5* mutant APA (Table 1). There were also no significant differences in age at adrenalectomy or tumor size between patients that harbored a *KCNJ5* mutant APA, compared to patients that harbored a *KCNJ5* wild-type APA (47 ± 12.6 vs. 46 ± 11.0 years old; 13.6 ± 4.47 vs. 13.0 ± 4.39 mm), though male patients in general tended to have adrenalectomy at an older age than females (49 ± 12.0 vs. 43 ± 10.2, *p* = 0.056; Table 2). Clinical attributes of pre-adrenalectomy and post-adrenalectomy were also not significantly different between patients with or without a *KCNJ5* mutant APA; though when compared by gender, pre-adrenalectomy and post-adrenalectomy serum creatinine levels, and post-adrenalectomy diastolic blood pressure were significantly lower in females (Table 2).

**Table 1:** Characterization of Malaysian cohort by the APA *KCNJ5* mutant status.

**Variables**	***KCNJ5* mutant**	***KCNJ5* wild-type**	***P-*value**
*Demography*	*n = 17*	*n = 37*	
Male:Female, *n* (% Female)	11:6 (23.1%)	17:20 (76.9%)	0.249
Malay:Chinese, *n* (% Chinese)	8:8 (33.3%)	18:16 (66.7%)	1.000
Age of adrenalectomy, y	47 ± 12.6	46 ± 11.0	0.679
Tumor diameter, mm	14 ± 4.5	13 ± 4.4	0.674
*Histological profile*	*n = 17*	*n = 37*	
CYP11B2, score	6 ± 2.5	6 ± 2.2	0.931
CYP17A1, score	7 ± 2.5	6 ± 3.6	0.560
*KCNJ5*, score	6 ± 2.6	6 ± 3.1	0.992
Active caspase 3, score	1 ± 2.2	1 ± 0.9	0.736
ZF-like cells, %	84 ± 27.7	83 ± 24.3	0.313
Atypical cells, %	0.04 ± 0.083	0.03 ± 0.067	0.277
Spironolactone bodies, count	0.4 ± 0.83	2.1 ± 9.26	0.327
*Pre-adrenalectomy attributes*	*n = 10*	*n = 19*	
SBP, mmHg	162 ± 32.3	149 ± 17.3	0.243
DBP, mmHg	95 ± 20.1	89 ± 15.7	0.418
Sodium, mmol/l	140 ± 2.7	140 ± 3.6	0.971
Potassium, mmol/l	2.7 ± 0.77	2.9 ± 0.70	0.308
Urea, mmol/l	4.4 ± 1.94	5.7 ± 2.19	0.124
Creatinine, μmol/l	84 ± 26.0	99 ± 35.1	0.308
Aldosterone, pg/ml	1,113 ± 1,694.8	609 ± 416.5	0.512
*Post-adrenalectomy attributes*	*n = 10*	*n = 19*	
SBP, mmHg	139 ± 19.1	128+14.1	0.089
DBP, mmHg	80 ± 12.2	79 ± 10.8	0.864
^*^Sodium, mmol/l	137 ± 3.5	137 ± 2.6	0.848
Potassium, mmol/l	4.5 ± 0.57	4.4+0.44	1.000
^*^Urea, mmol/l	6.9 ± 2.08	7.2 ± 2.31	0.694
^*^Creatinine, μmol/l	119 ± 46.0	117 ± 34.4	0.894

* *Data for one patient is loss to follow up*.

**Table 2:** Characterization of Malaysian cohort by gender.

**Variables**	**Male**	**Female**	***P-*value**
*Demography*	*n = 28*	*n = 26*	
*KCNJ5* WT:*KCNJ5* mutant, *n* (% mutant)	17:11 (64.7%)	20:6 (35.3%)	0.249
Malay:Chinese, *n* (% Chinese)	15:13 (54.2%)	11:11 (45.8%)	1.000
Age of adrenalectomy, y	49 ± 12.0	43 ± 10.2	0.056
Tumor diameter, mm	13 ± 4.2	14 ± 4.6	0.268
*Histological profile*	*n = 28*	*n = 26*	
CYP11B2, score	5 ± 2.4	7 ± 1.8	**0.010**
CYP17A1, score	6 ± 3.5	7 ± 3.2	0.440
*KCNJ5*, score	6 ± 3.0	5 ± 2.9	0.359
Active caspase 3, score	1 ± 1.86	0 ± 0.43	0.079
ZF-like cells, %	86 ± 23.6	81 ± 26.8	0.246
Atypical cells, %	0.04 ± 0.090	0.02 ± 0.044	0.768
Spironolactone bodies, count	1.0 ± 3.80	2.2 ± 10.44	0.712
*Pre-adrenalectomy attributes*	*n = 17*	*n = 12*	
SBP, mmHg	157 ± 27.7	148 ± 17.0	0.358
DBP, mmHg	94 ± 19.2	87 ± 13.5	0.258
Sodium, mmol/l	141 ± 3.5	139 ± 2.6	0.129
Potassium, mmol/l	2.8 ± 0.73	2.9 ± 0.72	0.471
Urea, mmol/l	5.2 ± 2.40	5.2 ± 1.88	0.996
Creatinine, μmol/l	108 ± 33.6	74 ± 18.1	**0.008**
Aldosterone, pg/ml	666 ± 334.6	948 ± 1603.6	0.370
*Post-adrenalectomy attributes*	*n = 17*	*n = 12*	
SBP, mmHg	136 ± 19.0	126 ± 11.4	0.189
DBP, mmHg	83 ± 8.4	74 ± 12.7	**0.038**
^*^Sodium, mmol/l	137 ± 2.8	137 ± 3.1	0.601
Potassium, mmol/l	4.4 ± 0.46	4.4 ± 0.52	0.963
^*^Urea, mmol/l	6.8 ± 2.03	7.6 ± 2.48	0.362
^*^Creatinine, μmol/l	132 ± 39.4	95 ± 21.2	**0.009**

* *Data for one patient is loss to follow up. Bold values indicates p < 0.05*.

### Histological Characteristics of *KCNJ5* Mutant APAs

**Figure 3:**
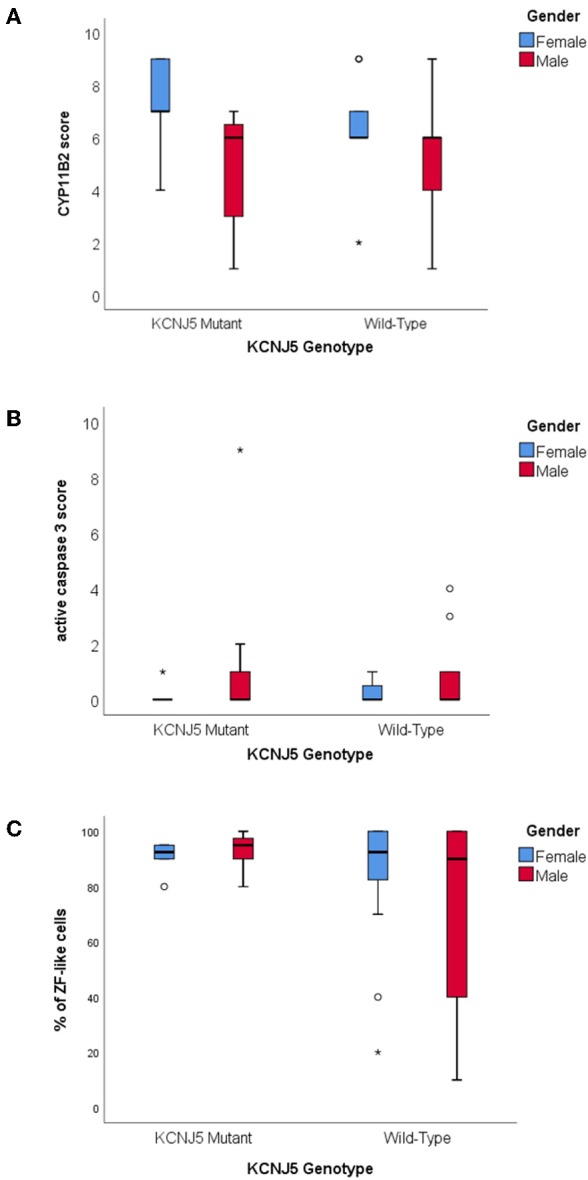
Histological characteristics of *KCNJ5* mutant APAs. **(A)** CYP11B2 protein expression, **(B)** active caspase 3 protein expression, and **(C)** percentage of ZF-like cells (i.e., cells with high cytoplasm: nucleus ratio), in *KCNJ5* mutant APAs grouped by gender.

There were no significant differences between CYP11B2, CYP17A1, *KCNJ5*, or active caspase 3 protein expression between *KCNJ5* mutant APAs compared to the wild-type. When compared by gender, APAs from a female patient had higher CYP11B2 expression (*U* = 220, *p* = 0.01, Figure 3A, Table 2) whereas APAs from a male patient had a trend to have higher active caspase 3 expression than APAs from a female patient (*U* = 280, *p* = 0.08, Figure 3B, Table 2). Similarly, there were no significant differences between the percentage of cells with ZF-like cell morphology (high cytoplasm: nucleus ratio), the percentage of atypical cells, and counts of spironolactone bodies in *KCNJ5* mutant APAs compared to the wild-type. However, of all histological parameters interrogated, only the percentage of cells with ZF-like cell morphology in the *KCNJ5* mutant and wild-type APAs had unequal variances [*F*_(1, 52)_ = 5.932, *p* = 0.02]. This is mainly driven by the bimodal distribution of the percentage of the ZF-like cell morphology in *KCNJ5* wild-type APAs caused by the large variances in wild-type APAs from male patients (Figure 3C and Supplementary Figure 2).

### Correlations of Histological Parameters

**Figure 4:**
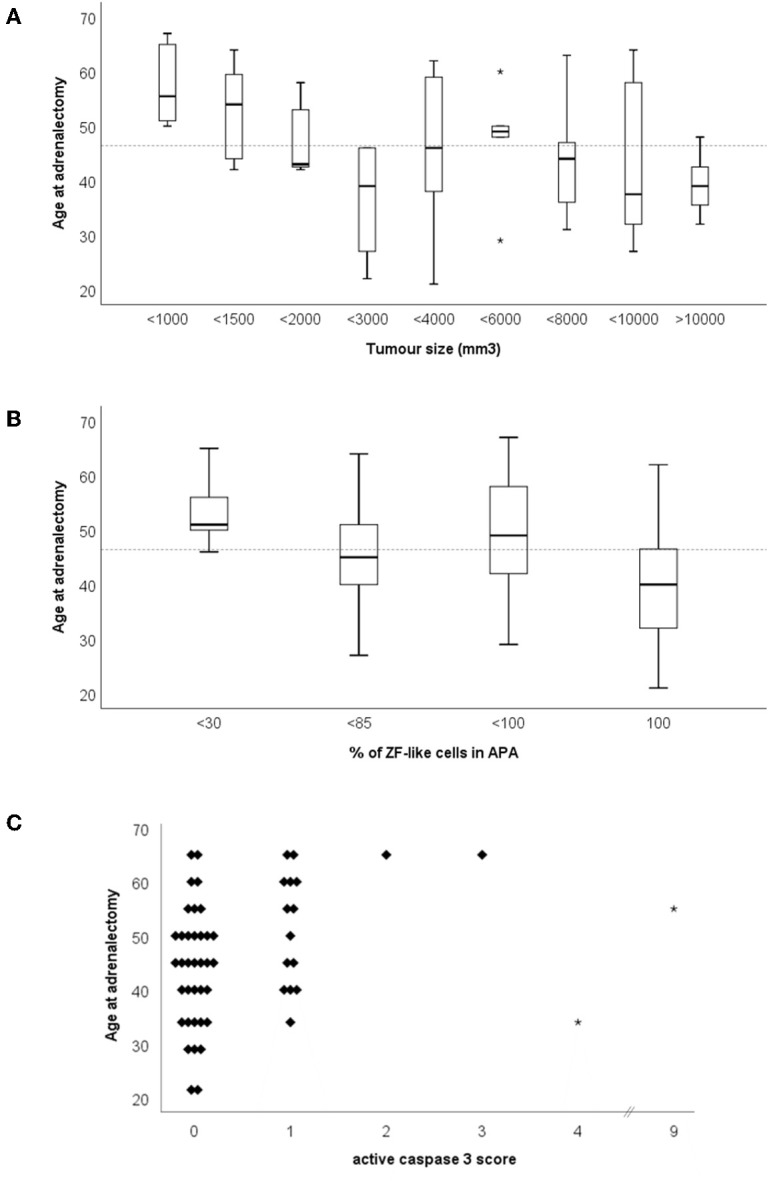
Age of adrenalectomy correlations with **(A)** tumor size, **(B)** percentage of ZF-like cells in APA (i.e., cells with high cytoplasm: nucleus ratio), and **(C)** active caspase three score. *Dotted line represents the mean age of adrenalectomy (46.4 years old).

The age of adrenalectomy correlated negatively with tumor size and the percentage of cells with ZF-like cell morphology in APA (Figure 4A, *r*_s_ = −0.475, *p* = 0.0003; and Figure 4B, *r*_s_ = −0.350, *p* = 0.01), and positively with active caspase 3 expression in APA (Figure 4C, *r*_s_ = 0.329, *p* = 0.015). The majority of tumors with a diameter <12.5 mm were adrenalectomized when the patients were above the mean age of adrenalectomy, whereas the majority of tumors with a diameter >20.0 mm were adrenalectomized when the patients were below the mean age of adrenalectomy. Interestingly, tumor size also weakly correlated positively with CYP17A1 expression in APA (Supplementary Figure 3A, *r*_s_ = 0.297, *p* = 0.03) and there was a trend of a correlation between tumor size and the percentage of cells with a ZF-like cell morphology in APA (*r*_s_ = 0.242, *p* = 0.08). The majority of tumors with <30% of ZF-like cells in APA were therefore adrenalectomized when the patients were above the mean age of adrenalectomy, whereas the majority of tumors with 100% ZF-like cells in APA were adrenalectomized when the patients were below the mean age of adrenalectomy. Between histological parameters, spironolactone body count correlated with CYP11B2 expression (Supplementary Figure 3B, *r*_s_ = 0.389, *p* = 0.004). To note, six of the 11 APAs with spironolactone bodies had <80% ZF-like cells, while APAs with >20 spironolactone body counts had <20% ZF-like cells.

## Discussion

The recommended treatment for a unilateral aldosterone-producing lesion that causes primary aldosteronism associated hypertension is adrenalectomy of the affected adrenal (35). Numerous studies performed on the excised tissues have found that a high prevalence of the *KCNJ5* mutation to be the cause of this pathology (18–20, 34). Although this study similarly showed *KCNJ5* mutant APAs to be common among Malaysian PA patients, the estimated prevalence of 37% is much lower than other Oriental cohorts that documented a prevalence of >50% (23, 27–33). The current population of Malaysia is comprised of 69.1% Bumiputera (of which the majority are Malays), 23.0% Chinese, 6.9% Indians and 1% other ethnicities (36). In this study, a similar number of Malay PA patients and Chinese PA patients were recruited, yet there were no significant differences between the number of Malay patients and the number of Chinese patients that had a *KCNJ5* mutant APA (31 vs. 33%).

IHC staining of tumor tissues has become an important tool to further characterize APAs (37). As such, four protein staining—CYP11B2, CYP17A1, active caspase 3, and KCNJ5, were used to characterize the *KCNJ5* mutant APAs. CYP11B2, also known as aldosterone synthase, is an enzyme that is essential for aldosterone synthesis as it is the sole enzyme responsible for the conversion of 11-deoxycorticosterone to corticosterone, to 18-hydroxycorticosterone, and finally to aldosterone. CYP17A1 has 17-alpha-hydroxylase and 17, 20 lyase activity which is needed to form the pre-cursors of cortisol, the main steroid produced physiologically in the ZF of the adrenal. Active caspase 3 is a protein that has been reported to play a role in cell apoptosis to the extent that alteration of the caspase 3 gene, CASP3, is reported to promote human tumorigenesis (38). *KCNJ5* protein expression is not only of interest, as somatic mutations in the gene is common in APAs, but also as it is more highly expressed in the ZG, the physiological zone in the adrenal that produces aldosterone, than the ZF (13).

In this study, there were no significant differences in the expression of CYP11B2, CYP17A1, active caspase 3, or KCNJ5 between the *KCNJ5* mutant and wild-type APAs. Similarly, the percentage of cells with a ZF-like cell morphology (high cytoplasm: nucleus ratio), the percentage of atypical cells, and counts of spironolactone bodies in *KCNJ5* mutant APAs compared to the wild-type, showed no significant difference. Some studies in the Caucasian cohorts had found *KCNJ5* mutant APAs to have a significantly lower expression of the KCNJ5 protein and a ZF-like cell profile (25, 37, 39, 40) which is in agreement with the infrequent *KCNJ5* mutations observed in the ZG-like APCCs and nodular lesions (16, 17). The lack of the *KCNJ5* genotype correlation with the ZF-like cell profile in this study is arguably more likely due to a difference in the Malaysian “*KCNJ5* wild-type” APA cohort rather than due to a true difference between Malaysian *KCNJ5* mutant APAs and Caucasian *KCNJ5* mutant APAs. In this Malaysian cohort only five APAs had a diameter <10 mm^3^, likely due to our center's low success rate in adrenal vein sampling (<25%) preventing adenoma's not seen by CT-scan from undergoing adrenalectomy. Thus, our “*KCNJ5* wild-type” APA cohort may contain fewer *CACNA1D* mutant APAs as these APAs have been reported to be smaller in size and angiotensin II-responsive, making them harder to be diagnosed (18–20, 41). These *CACNA1D* mutant APAs have been reported to be more common among males and to have a non-ZF-like (i.e., ZG-like) cell profile (11, 19, 20). Therefore, if we were to assume that our “*KCNJ5* wild-type” cohort is missing smaller ZG-like *CACNA1D* mutant APAs, the results from this Malaysian cohort is in agreement with previous reports of *KCNJ5* mutant APAs with a ZF-like cell profile, as almost all the cells in *KCNJ5* mutant APAs were ZF-like, whereas the percentage of ZF-like cells in *KCNJ5* wild-type APAs varied especially in males (Figure 1C and Supplementary Figure 2).

The majority of studies documenting the *KCNJ5* genotype of APAs reported that patients harboring a *KCNJ5* mutant APA were more commonly females, adrenalectomized at a younger age, with a larger tumor size than those harboring a *KCNJ5* wild-type APA (18–25, 34, 41). Though, some studies in Oriental cohorts had found no gender bias, as male and female APAs had similar *KCNJ5* mutation rates (23, 29–31, 33). In this study, males more commonly harbored a *KCNJ5* mutant APA compared to females (39% vs. 23%) and there were no significant differences in age at adrenalectomy or tumor size between patients with a *KCNJ5* mutant APA compared to patients with a *KCNJ5* wild-type APA. However, compared to female patients, male patients in general tended to have adrenalectomy at an older age (49 + 12.0 vs. 43 + 10.2; *p* = 0.056) and tended to have a higher expression of the apoptosis marker active caspase 3 (*U* = 280, *p* = 0.08). Altogether the findings suggest that perhaps undergoing adrenalectomy at a younger age and having a larger tumor size is reflective of patients' gender rather than the *KCNJ5* genotype of the APA. The difference in adrenalectomy age would also explain the significantly lower post-adrenalectomy diastolic blood pressure in females. It is worth noting that the primary female hormone, estrogen, is well-known to play a role in the development and malignant progression of multiple cancers, and that estrogen receptors located in both the nucleus and the cytoplasm of tumor cells regulates genes involved in cell survival and proliferation (42–44). We had previously noted that the estrogen related receptor beta gene, ESRRB, was 3-fold up-regulated in APAs compared to their adjacent normal adrenals, and in this study, APAs from female patients had more CYP11B2 expression than APAs from male patients [Figure 3A; (25)]. Concurringly, during murine embryogenesis the ESRRB protein is expressed in the adrenal primordium, supporting the role of estrogen in the development and proliferation of adrenal cells (45).

Correlation of histological parameters in this study showed that age of adrenalectomy negatively correlated with tumor size and the percentage of ZF-like cells in APA. There was also a weak positive correlation between the ZF enzyme CYP17A1 expression in APA and tumor size, and a trend for a positive correlation between the percentage of ZF-like cells in APA and tumor size. Conjointly our results suggest that APAs with a ZF-like profile (either based on steroid enzyme expression or cell morphology) tends to be larger and thus probably diagnosed faster resulting in the treatment, adrenalectomy, to occur in the patient at a younger age. Interestingly, we did not find spironolactone bodies to be common in ZF-like APAs, as APAs with >20 spironolactone body counts had <20% ZF-like cells. Association between spironolactone bodies and ZG or ZG-like APAs has previously been documented (46, 47). Perhaps this finding is due to ZG-like APAs being harder to diagnose and therefore the patient's hypertension is treated with spironolactone for longer, compared to patients with a ZF-like APA.

As this was a retrospective study, the mutation analysis was limited to patients for whom APA tissues were available and that were of good quality. Therefore, whether this cohort truly represents the prevalence of *KCNJ5* mutant APAs in a Malaysian population is still questionable as there would be more usable tissues for larger APAs. Moreover, due to reliance on CT-scan findings for a decision of adrenalectomy, when adrenal vein sampling is unsuccessful, the findings from this study could simply be a case of a comparison between “low-hanging fruits”—i.e., the comparison of *KCNJ5* mutant APAs among large APAs. Nevertheless, despite these limitations, our findings do speculatively suggest that the phenotype previously connected with *KCNJ5* mutant APAs may actually be the phenotype of APAs adrenalectomized from female PA patients. Further investigation through a multicenter prospective study, recruiting a larger number of Malaysian patients, is warranted.

## Data Availability Statement

This manuscript contains previously unpublished data. The data that support the findings of this study are available from the corresponding author, EA, upon reasonable request.

## Ethics Statement

This study was carried out in accordance with the recommendations of the local research ethics committee of the National University of Malaysia Medical Center. As only somatic mutations in the hotspot of *KCNJ5* was interrogated in archived tissue, and results were not presented for individuals, individual informed consent of subjects were not taken. The protocol was approved by the local research ethics committee of the National University of Malaysia Medical Center under the project code FF-2016-161.

## Author Contributions

EA, NK, RM, AJ, and NS contributed to the conception and design of the research. SM, MM, and GT contributed to the acquisition of the work. EA and SM contributed to the analysis and interpretation of data for the work.

### Conflict of Interest

The authors declare that the research was conducted in the absence of any commercial or financial relationships that could be construed as a potential conflict of interest.
