# Supplementary material for: Prevalence and Histopathological Characteristics of KCNJ5 Mutant Aldosterone-Producing Adenomas in a Multi-Ethnic Malaysian Cohort
Source: Front Endocrinol (Lausanne). 2019 Oct 4;10:666. doi: 10.3389/fendo.2019.00666 (PMC6787170; doi:10.3389/fendo.2019.00666)
Supplement: Supplementary file 1 [file Data_Sheet_1.docx]

Supplementary Material

# Supplementary Method

## Immunohistochemistry (IHC) Staining Method

IHC staining was performed on tissue sections using the protocol from EnVision™ FLEX+, Mouse, High pH (Dako, Denmark) according to manufacturer’s instructions. In brief, primary antibodies were diluted to optimal concentration using Antibody Diluent, Dako REAL^TM^. Washing steps between each reagent were performed using EnVision^TM^ FLEX Wash Buffer 20x diluted to a 1X working solution with deionized water. The 1X DAB-containing Substrate Working Solution was prepared by diluting the 50X concentrated EnVision^TM^ FLEX DAB+ Chromogen with Envision^TM^ FLEX Substrate Buffer. Tissue blocks were sectioned approximately 4 µm thickness and mounted on standard adhesive glass slides. The slides were left to be air-dried in room temperature overnight. The tissue slides were then incubated on a hot plate at 60°C for 1 hour. An initial deparaffinization and pre-treatment step was performed in the Dako PTLink using the EnVision^TM^ FLEX Target Retrieval Solution, High pH followed by cooling at room temperature for 20 minutes and rinsed with running tap water for 3 minutes. The slides were subsequently incubated with EnVision^TM^ FLEX Peroxidase-Blocking Reagent for 5 minutes followed by washing step. Slides were then incubated with primary antibody at room temperature then followed by incubation with EnVision^TM^ FLEX/HRP for 20 minutes. Sections were then incubated with 1X DAB-containing Substrate Working Solution for 10 minutes. The slides were then counterstained with Hematoxylin 2 (Product Code #7231, ThermoScientific, USA) for 5 seconds after the procedures have been completed followed by dehydration step with increasing alcohol solutions ( 80%, 90%, 100% and 100%) and 2-times Xylene. Finally, the slides were mounted and cover-slipped using DPX mounting medium (Product Code #100579, Merck Milipore, Germany). To prevent diagnostic drift and chronological bias, a control adrenal tissue was performed with every batch of slides scored of which if scored differently from 1st batch, would flag the scores of that batch for review and comparison with 1st batch.

# Supplementary Figures and Tables

## Supplementary Figures

(a)


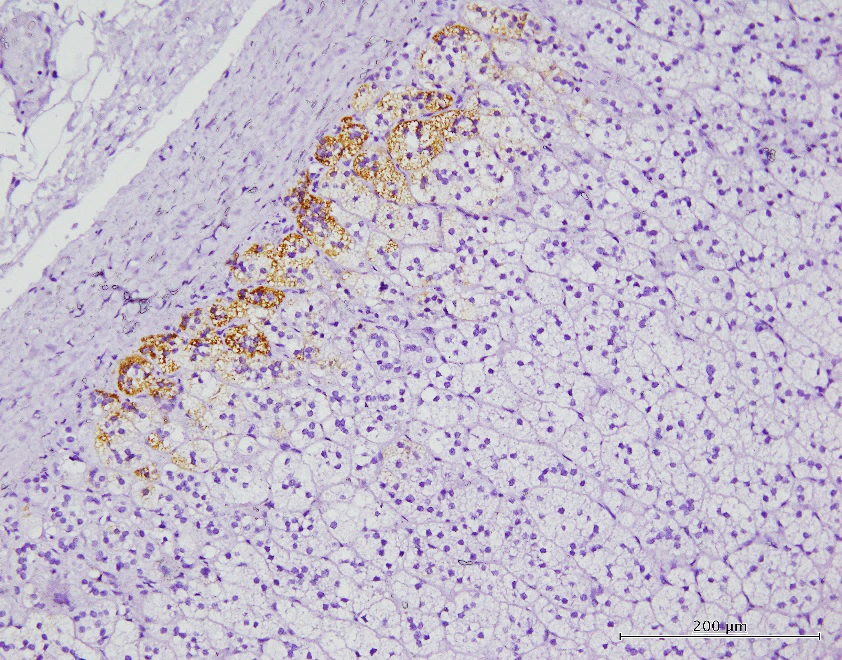


(b)


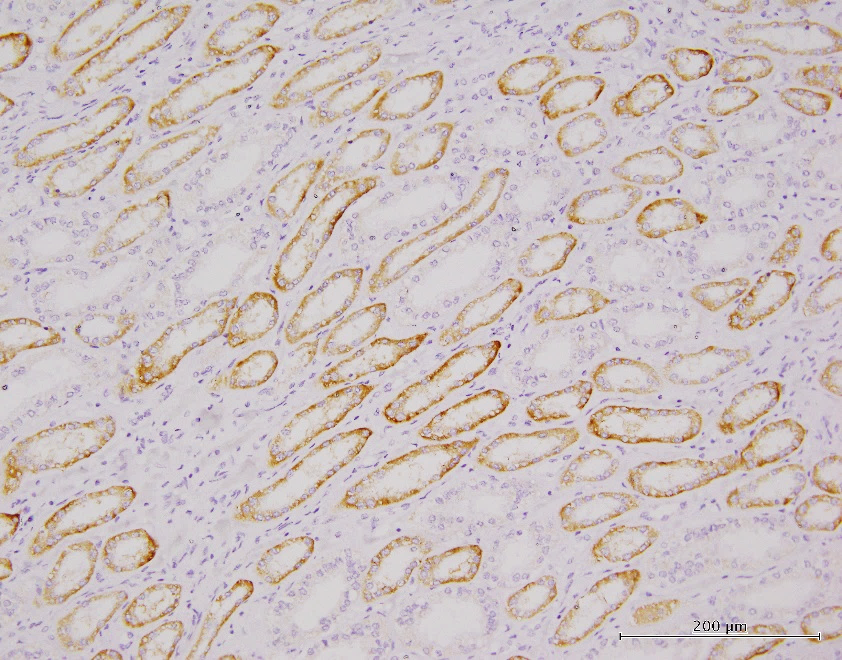


(c)


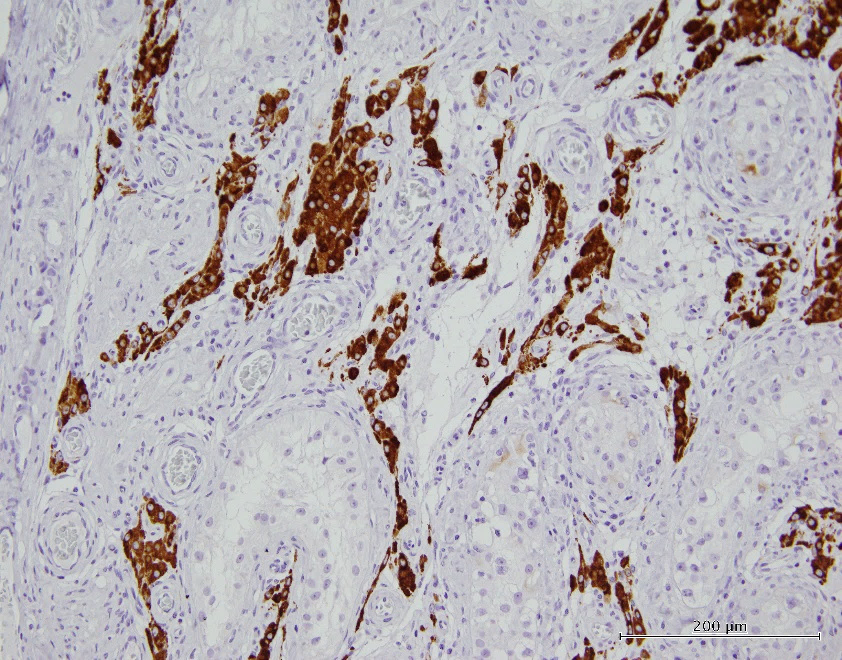


(d)


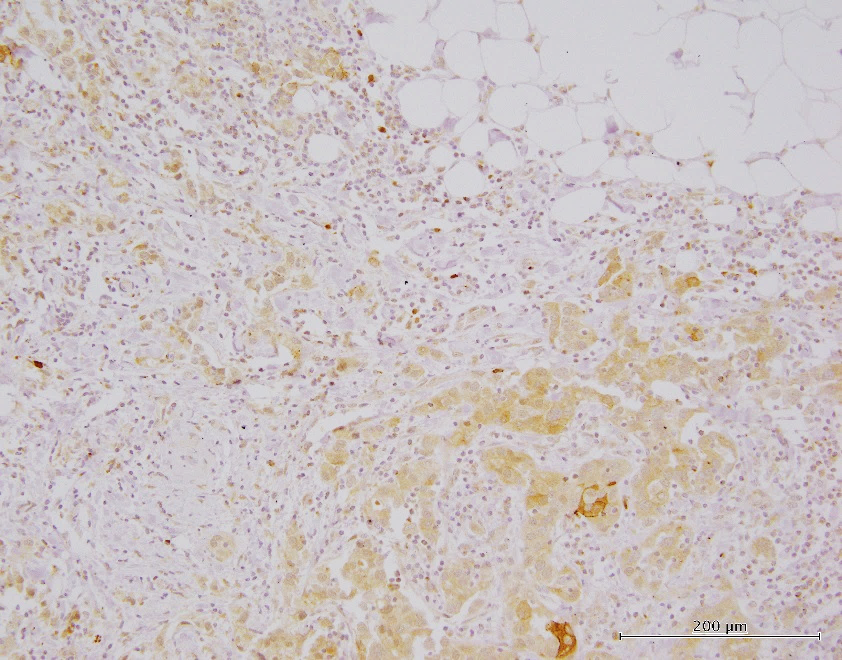


**Supplementary Figure 1.** Specific and selective positive control tissue staining of (a) CYP11B2 in adrenal, (b) KCNJ5 in kidney, (c) CYP17A1 in testis, and (d) active caspase 3 in breast carcinoma.


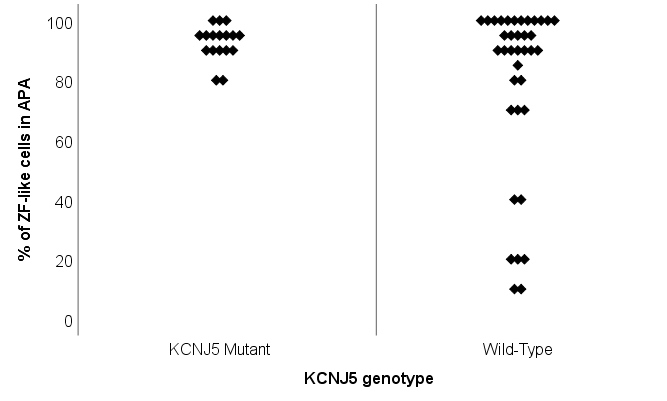

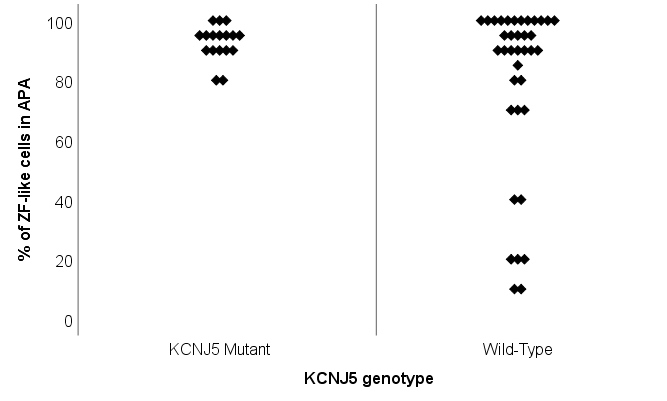

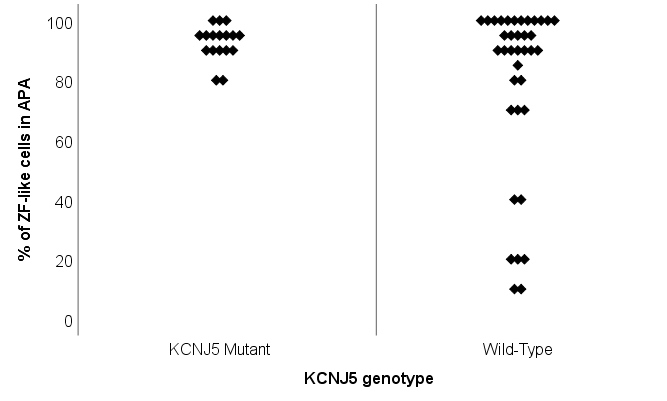

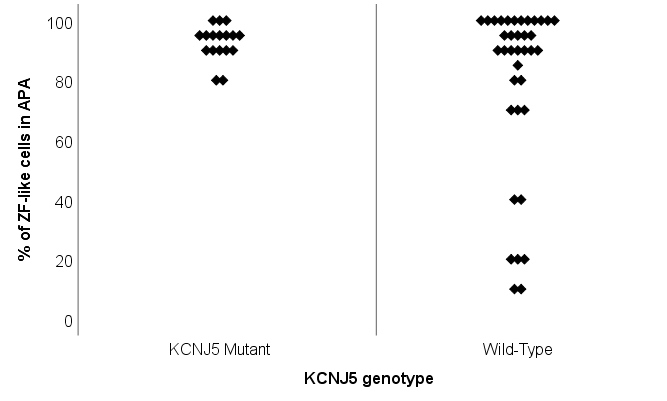


**Supplementary Figure 2.** Bimodal distribution of percentage of ZF-like cells in KCNJ5 wild type APAs.

(a)


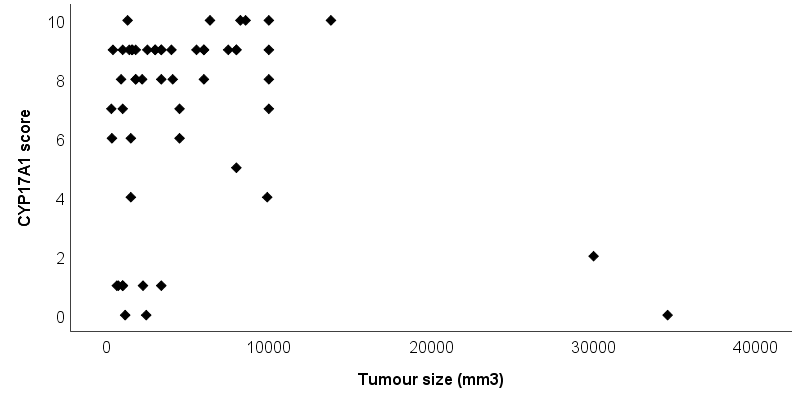


(b)


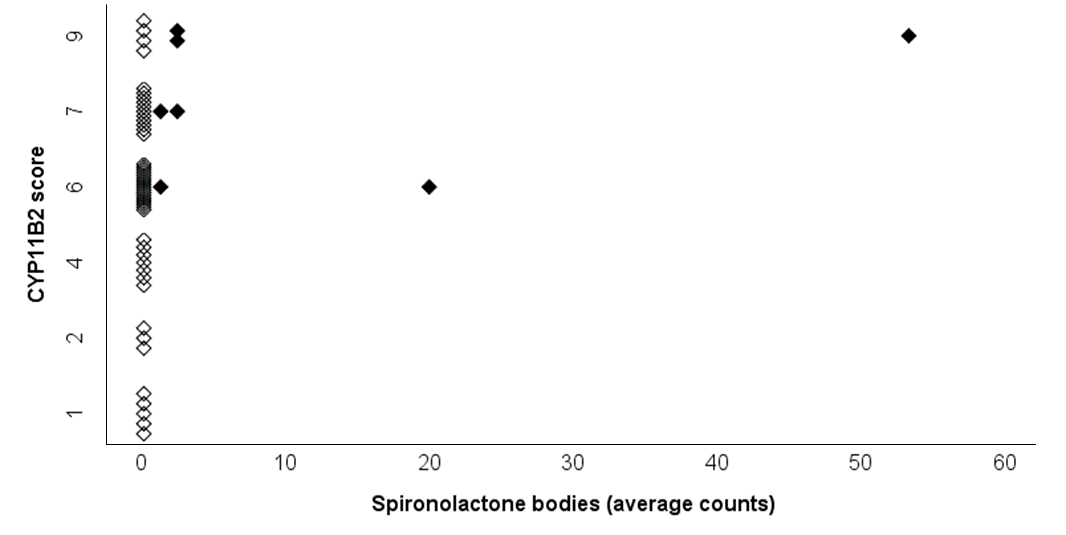


**Supplementary Figure 3.** Correlations of histological parameters between (a) CYP17A1 score and tumour size, and (b) CYP11B2 score and the average spironolactone bodies counts.

## Supplementary Tables

**Supplementary Table 1.** List of primary antibodies and parameters used for IHC staining.

| Primary Antibody | Product Code # | Antibody Source | Biological Source  Clone | Primary Antibody Dilution | Primary Antibody Incubation Time | Pre-treatment Conditions (Temperature / Time) |
| --- | --- | --- | --- | --- | --- | --- |
| anti-KCNJ5 | HPA017353 | Sigma-Aldrich, USA | Rabbit  Polyclonal | 1:100 | 20 minutes | 95°C / 20 minutes |
| anti-CYP17A1 | *NA - Gift | Celso Gomez-Sanchez | Rabbit  Polyclonal | 1:200 | 20 minutes | 95°C / 20 minutes |
| anti-CYP11B2 | *NA - Gift | Celso Gomez-Sanchez | Mouse Monoclonal | 1:100 | 60 minutes | 98°C / 30 minutes |
| anti-Active Caspase 3 | G748A | Promega  USA | Rabbit  Polyclonal | 1:250 | 30 minutes | 110°C / 30 minutes |

*NA, not applicable

**Supplementary Table 2.** The scoring table used to quantify the IHC staining.

| Score | Staining pattern |
| --- | --- |
| 0 | No membranous and no cytoplasmic staining (negative expression) |
| 1 | Some staining in <20% of cells |
| 2 | Weak to moderate membrane/cytoplasmic staining in >20%<50% of cells |
| 3 | Weak to moderate membrane and cytoplasmic staining in >20%<50% of cells |
| 4 | Weak to moderate membrane/cytoplasmic staining in majority of cells |
| 5 | Weak to moderate membrane and cytoplasmic staining in majority of cells |
| 6 | Strong staining in >20%<50% of cells |
| 7 | Strong membrane/cytoplasmic staining in majority of cells |
| 8 | Strong membrane and cytoplasmic staining in majority of cells |
| 9 | Strong membrane/cytoplasmic staining in >90% of cells |
| 10 | Strong membrane and cytoplasmic staining in >90% of cells |

**Supplementary Table 3.** Primer sequence used for *KCNJ5* PCR and sequencing. *KCNJ5* PCR was performed on every sample twice using different primer pairs.

| Forward primer 1 | GGA TTC CTT GTG TTG AAA ACC |
| --- | --- |
| Reverse primer 1 | TCT TGG GCT GGC TGA TCT T |
| Forward primer 2 | CAA CTT GCT CGT CTT CAC CA |
| Reverse primer 2 | GAG GGT CTC CGC TCT CTT CT |

**Supplementary Table 4.** Parameters used for *KCNJ5* PCR.

| Primers concentration | 0.5 µM |
| --- | --- |
| DNA concentration | 200 ng |
| Enzyme activation | 95˚C for 10 minutes |
| Denaturing temperature | 96˚C for 3 seconds |
| Annealing temperature | 55-57˚C for 3 seconds |
| Extension temperature | 68˚C for 5 seconds |
| PCR cycles | 35x  (Denaturing, Annealing, Extension step) |
| Final extension temperature | 72˚C for 10 seconds |
